# Dynamic alterations of spontaneous neural activity in post-stroke aphasia: a resting-state functional magnetic resonance imaging study

**DOI:** 10.3389/fnins.2023.1177930

**Published:** 2023-05-11

**Authors:** Luyao Xu, Hongchun Wei, Zhongwen Sun, Tongpeng Chu, Min Li, Ruhui Liu, Li Jiang, Zhigang Liang

**Affiliations:** ^1^Department of Neurology, Yantai Yuhuangding Hospital Affiliated to Qingdao University, Yantai, Shandong, China; ^2^Department of Radiology, Yantai Yuhuangding Hospital Affiliated to Qingdao University, Yantai, Shandong, China

**Keywords:** post-stroke aphasia, resting-state functional magnetic resonance imaging, dynamic amplitude of low-frequency fluctuation, brain networks, clustering

## Abstract

**Background and purpose:**

The dynamic alterations in spontaneous neural activity of the brain during the acute phase of post-stroke aphasia (PSA) remain unclear. Therefore, in this study, dynamic amplitude of low-frequency fluctuation (dALFF) was applied to explore abnormal temporal variability in local functional activity of the brain during acute PSA.

**Materials and methods:**

Resting-state functional magnetic resonance imaging (rs-fMRI) data from 26 patients with PSA and 25 healthy controls (HCs) were acquired. The sliding window method was used to assess dALFF, with the k-means clustering method used to identify dALFF states. The two-sample *t*-test was applied to compare differences in dALFF variability and state metrics between the PSA and HC groups.

**Results:**

(1) In the PSA group, greater variance of dALFF in the cerebellar network (CBN) and left fronto-temporo-parietal network (FTPN) was observed. (2) Three dALFF states were identified among all subjects. States 1 and 2 were identified in the PSA patients, and the two dALFF states shared a similar proportion. Moreover, the number of transitions between the two dALFF states was higher in the patients compared with that in HCs.

**Conclusion:**

The results of this study provide valuable insights into brain dysfunction that occurs during the acute phase (6.00 ± 3.52 days) of PSA. The observed increase in variability of local functional activities in CBN and left FTPN may be related to the spontaneous functional recovery of language during acute PSA, and it also suggests that cerebellum plays an important role in language.

## Introduction

Stroke is the most common cause of aphasia, with approximately 21–38% of all strokes resulting in aphasia (Engelter et al., 2006). Post-stroke aphasia (PSA) is caused by cerebrovascular disease (especially an occlusion within the region of the left middle cerebral artery), which leads to damage in cerebral language-related areas or disturbance of the language network (Fridriksson et al., 2018; Klingbeil et al., 2019). Patients with PSA typically manifest a poor ability to communicate. This adversely affects treatment, while also reducing their ability to engage socially and enjoy a good quality of life (El Hachioui et al., 2013). Expert consensus prompted to designate the patients with PSA in acute stage have some degree of spontaneous functional recovery of language. However, the mechanism of this spontaneous functional recovery of language is still unclear, and the recovery mechanism of PSA is still in the exploring. Therefore, a comprehensive understanding of the recovery mechanism(s) involved is essential, this will also help inform clinical treatment strategies.

Over the last several years, resting-state functional magnetic resonance imaging (rs-fMRI) has been used as a tool for uncovering underlying brain recovery mechanism(s) of PSA (Klingbeil et al., 2019). As a result, alterations in functional activities of brain regions and functional connectivity of brain networks have been observed following PSA (Stockert et al., 2020; Zhang et al., 2021). Regarding functional recovery of language after PSA, it is dependent on both functional reorganization of the left hemisphere’s spare region and functional compensation of right language-homologous brain regions (Sims et al., 2016; Robson et al., 2017). When small lesions are present in language-related regions of the brain, functional recovery of language may depend on the extent of functional reorganization that occurs in perilesional regions (Hartwigsen and Saur, 2019). Conversely, when large lesions are present, the remaining language-related regions are insufficient to support functional recovery of language, making recovery more dependent on right language-homologous brain regions (Di Pino et al., 2014). In some studies, PSA has been characterized by a three-phase model of dynamic functional organization (Stockert et al., 2020). For example, decreased brain activation in the left hemisphere is observed in the acute phase; then a large increase in activation in the perilesional cortex and bilateral domain-general networks is observed in the subacute phase; and finally, normalization of activation in the left-hemispheric language areas occurs in the chronic phase.

It should be noted that many of the previously employed research methods of PSA are static. However, the brain dynamically integrates stimuli over time (Allen et al., 2014). Thus, static methods may not detect dynamic changes in interaction patterns (Avena-Koenigsberger et al., 2017). Conversely, dynamic methods have the potential to effectively capture dynamic characteristics of brain activity. Amplitude of low-frequency fluctuation (ALFF) is a method which can reflect the activity of spontaneous neurons in local brain regions (Hoptman et al., 2010). dALFF is an extension of ALFF, and it quantifies temporal variability of brain functional activity to observe internal changes. In recent years, some neurological and psychiatric diseases have been found to exhibit abnormal functional activities via dALFF, such as parkinson’s disease (Zhang et al., 2019), obsessive–compulsive disorder (Liu et al., 2021), and depression (Dong et al., 2022). However, to date, no study has applied dALFF to PSA. Furthermore, previous studies of PSA have largely focused on subacute and chronic PSA, yet rarely on acute PSA. Considering that the mechanism of spontaneous functional recovery of language in acute PSA is unclear, the aim of this study was to explore alterations in the dynamic properties of brain activity during acute PSA by observing dALFF variance. These data can potentially provide novel insights into the recovery mechanism(s) of PSA and better guide treatment.

## Materials and methods

### Participants

Twenty-six right-handed patients diagnosed with PSA and twenty-five age-, gender-, and education-matched healthy subjects were recruited. In this observational single-center study that was approved by the IRB of Yantai Yuhuangding Hospital Affiliated to Qingdao University (IRB no. 2022-173), all subjects provided written informed consent prior to their enrollment.

### Inclusion and exclusion criteria for participants

All of the PSA patients (*n* = 26) and age-, gender-, and education-matched healthy subjects (HCs; *n* = 25) were right-handed [assessed with the Edinburgh Handedness Inventory (Oldfield, 1971)], native Chinese speakers, and were aged more than 18 years and less than 80 years. Inclusion criteria were: (1) an aphasia diagnosis based on the Western Aphasia Battery (WAB) and Aphasia Quotient (AQ) < 93.8; (2) at the first onset of stroke, the lesion was confirmed to be located in the left hemisphere by cranial computed tomography or MRI; (3) the duration of aphasia was 1–14 days; (4) the patients are able to cooperate with a rs-fMRI scan; and (5) the education level of the patient was elementary school or above. Exclusion criteria were: (1) based on the WAB and AQ > 93.8; (2) speech, reading, or writing impairment due to severe damage to sensory and motor organs, such as hearing, vision, articulation, and writing; (3) recurrent stroke, cerebellar, or brainstem stroke; (4) those with a history of stroke, depression, neurological or psychiatric disorders; (5) subjects who were unconscious and unable to cooperate with a rs-fMRI scan or complete the scale assessment; (6) substance abuse that could interfere with cognitive function; and (7) contraindications to MRI. Inclusion criteria for the HCs were: (1) no previous history of stroke, depression, neurological or psychiatric disorders; (2) no language disturbance or cognitive impairment; (3) able to complete an assessment of the WAB-AQ scale and AQ > 93.8; and (4) able to complete a rs-fMRI scan.

### Assessment of aphasia

All participants underwent a comprehensive evaluation, including medical history, neurological examination, and neuroimaging. Aphasia was diagnosed according to the WAB assessment (Shewan and Kertesz, 1980). The latter surveys six areas of functioning: spontaneous speech, auditory comprehension, repetition, naming, reading, and writing. The AQ plays an important role in the WAB. In normal-speaking adults, their AQ values range from 98.4 to 99.6. An AQ value <93.8 is classified as aphasia (Ren et al., 2019).

### Magnetic resonance imaging data acquisition

MRI was performed using a 3.0 T MR scanner (GE 750 W, GE Healthcare, USA) with a standard eight-channel head coil. Resting-state blood oxygenation level-dependent MR images were acquired using the following parameters: TR/TE = 2000 ms / 30 ms, FA = 90°, FOV = 224 mm × 224 mm, slice number = 36, voxel size = 3.5× 3.5 × 3.0 mm3, slice thickness = 3 mm, and 175 volumes. Participants were asked to close their eyes, keep quiet, and avoid any head movement during the scan.

### rs-fMRI data preprocessing

Preprocessing of rs-fMRI data was conducted using DPARSF (a data processing and analysis tool for resting-state brain images^1^) (Yan et al., 2016). The first ten time-points were removed to acquire a rs-fMRI signal in a steady state. Slice timing and head movements were carried out on the remaining 165 time-points to eliminate differences. The resulting data were spatially normalized to the standard Montreal Neurological Institute template. Each voxel was resampled to 3 mm × 3 mm × 3 mm. The multiple linear regression method was used to regress some covariates (e.g., Friston-24 head-motion parameters, white matter signal, and cerebrospinal fluid signal). A 6-mm half-width smoothing kernel was used to spatially smooth images. Linear trend and band-pass filtering (0.01–0.08) were performed to remove signal drift and physiological noise. Participants with head motion >2.0 mm and rotation >2.0° were excluded, which included two PSA patients and one healthy subject.

### Feature framework of dALFF

**Figure 1:**
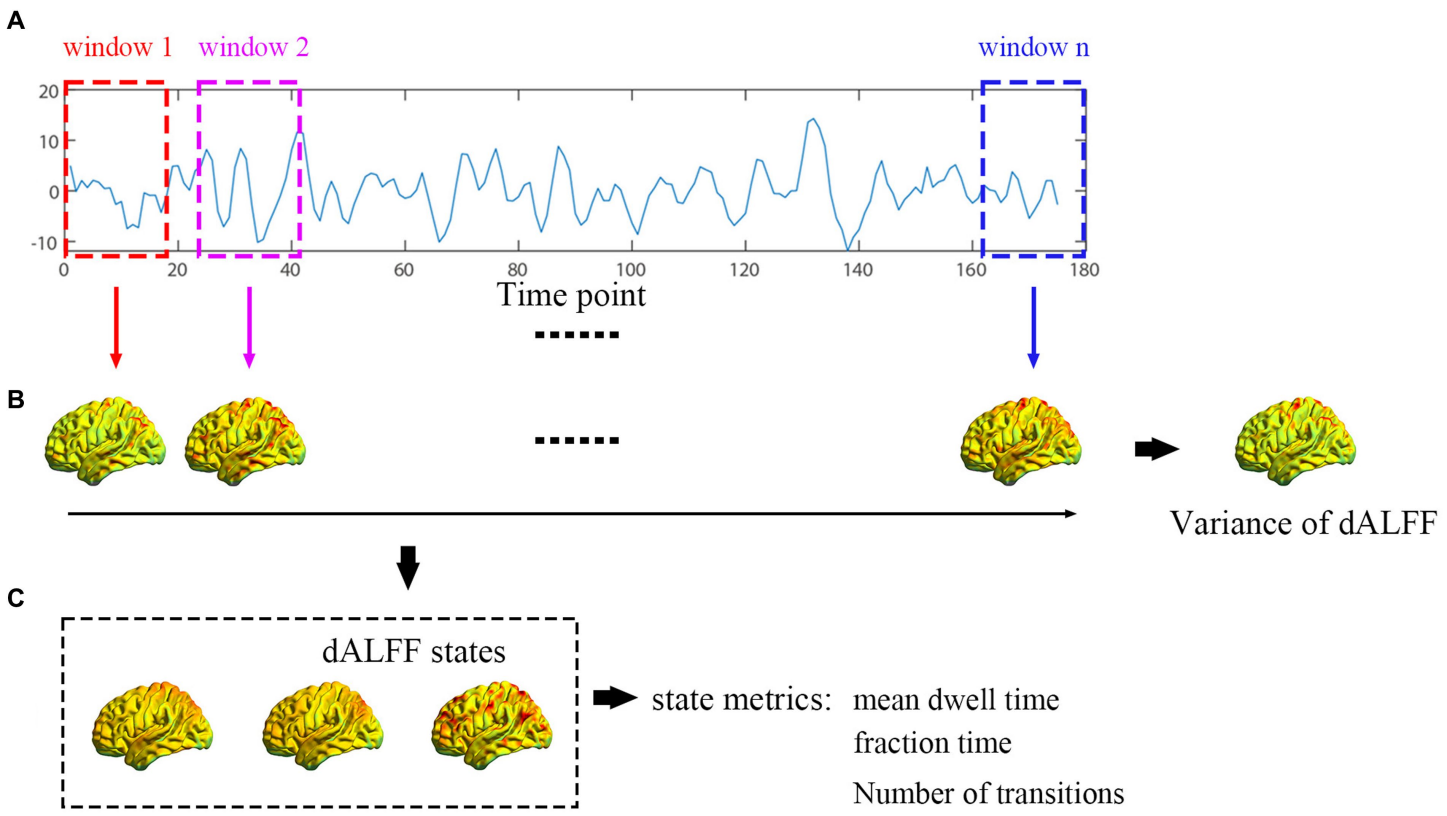
Flowchart for the exploration of dALFF conducted. **(A)** Each participant’s time point was collected and divided by the fixed length of the time window. **(B)** dALFF variability was computed. **(C)** dALFF clustered to three states according to the k-means cluster method.

The flowchart shown in Figure 1 includes three steps. (a) Collection of participants’ data; the fixed length of time window was acquired according to the sliding window method. (b) Variance values of dALFF were calculated. (c) dALFF variance values were clustered into three states.

### Analysis of dALFF variability

Analysis of dALFF was processed with the DynamicBC toolkit as implemented in Matlab (r2021a, MathWorks) (Liao et al., 2014). The sliding window parameter is important for capturing dynamic, spontaneous neural activity. For example, selection of a short window length may increase the risk of capturing misleading fluctuations, while selection of a long window length may not sufficiently capture dynamics (Li et al., 2019). The minimum window length should also not be less than 1/fmin. In the present study, a window size of 40 TRs and a sliding step of 1TR were selected to calculate dALFF variability. The calculation procedure included the following steps: (1) The time series of each subject was divided into 136 windows, then a set of ALFF maps were acquired by calculating the ALFF value in each window; (2) To estimate the power spectrum, each window data was first transformed to a frequency domain by using fast Fourier transform; (3) The square root of each frequency of all power spectra in the range of 0.01–0.08 Hz was computed to acquire an ALFF map, then the ALFF value was divided by the mean value of the whole brain to acquire a standardized ALFF map; (4) A dALFF map was acquired by connecting the ALFF values of all windows for each subject; (5) Temporal variability of ALFF was assessed by computing variance; and (6) Finally, for each subject, the dALFF variability of each voxel was further converted to z-scores.

### Clustering analysis

The dALFF values for all participants were subjected to a K-means algorithm to acquire the occurrence state of dALFF. The k-means algorithm aggregates information with similarities into “k” groups, thereby ensuring that the sum of squares within clusters is minimal (Zhang et al., 2018). The Manhattan distance (L1 distance) was used as a similarity measure in clustering. According to previous research (Allen et al., 2014), we used the subject exemplars as a subset of windows with local maxima in dALFF variance to perform k-means clustering with varying numbers of clusters k (2–10). Three was the optimal number of clusters based on the Davies-Bouldin Index (Vergara et al., 2020). The resulting three cluster centroids were regarded as starting points to cluster all of the dALFF data into three clusters. Finally, three states of dALFF were identified between the two groups. To analyze differences, the following parameters were examined: (1) mean dwell time (MDT): average number of consecutive windows belonging to a state; (2) fraction of spent time (F): number of windows per state; and (3) number of transitions (NT): the total number of transitions from one clustering state to another.

### Validation analysis

Validation analyses with different window lengths (30TRs and 40 TRs) and step size (1 TR and 2 TRs, respectively) were conducted to assess the reliability of our results.

### Statistical analysis

SPSS 26.0 software was used to perform statistical analyses. The non-parametric Mann–Whitney *U*-test was applied to assess between-group differences with respect to demographic variables, including age and education level. The Chi-squared test was applied to assess between-group differences with respect to categorical variables (e.g., gender). What’s more, *p* < 0.05 was statistically significant.

We completed the following analyses in DPABI version V6.1_220101. The two-sample *t*-test was applied to between-group comparisons of dALFF variability, with age, gender, and educational level were included as covariates. To reduce errors, the statistical level was set at *p* < 0.001 and corrected with Gaussian random field (GRF). The GRF is a relatively strict multi-comparison correction method, it can reduce the occurrence of false positive rate and has widely been used in many fMRI studies (Li et al., 2018). Two-sample *t*-test was performed to analyze the differences of state properties.

## Results

### Demographic and clinical characteristics

Demographic and clinical information for the cohort of this study summarized in Table 1. There were no significant differences observed between the PSA and HC groups with respect to age (*z* = −1.697, *p* = 0.090), gender (*χ*^2^ = 0.943, *p* = 0.331), or level of education (*z* = −1.325, *p* = 0.185).

**Table 1:** Demographic and clinical information for the study cohort.

Characteristics	Aphasia (*n* = 32)	HC (*n* = 32)	Aphasia vs. HC
	Mean ± SD	Mean ± SD	*p*-values
Handedness (left/right)	0/26	0/25	–
Gender	16 M / 10 F	12 M / 13 F	0.331
Age (y)	63.65 ± 16.32	59.24 ± 13.85	0.090
Education (y)	10.54 ± 2.58	11.48 ± 2.66	0.185
Time post-stroke (days)	6.00 ± 3.52	–	–
WAB-AQ scores		–	–
Spontaneous speech score	10.15 ± 6.17	–	–
Auditory comprehension score	144.00 ± 69.84	–	–
Repetition score	55.23 ± 33.27	–	–
Naming score	57.73 ± 32.92	–	–
AQ	57.07 ± 30.48	–	–

M, mean; SD, standard deviation; WAB-AQ, Western Aphasia Battery-Aphasia Quotient.

### Differences in dALFF variability

**Figure 2:**
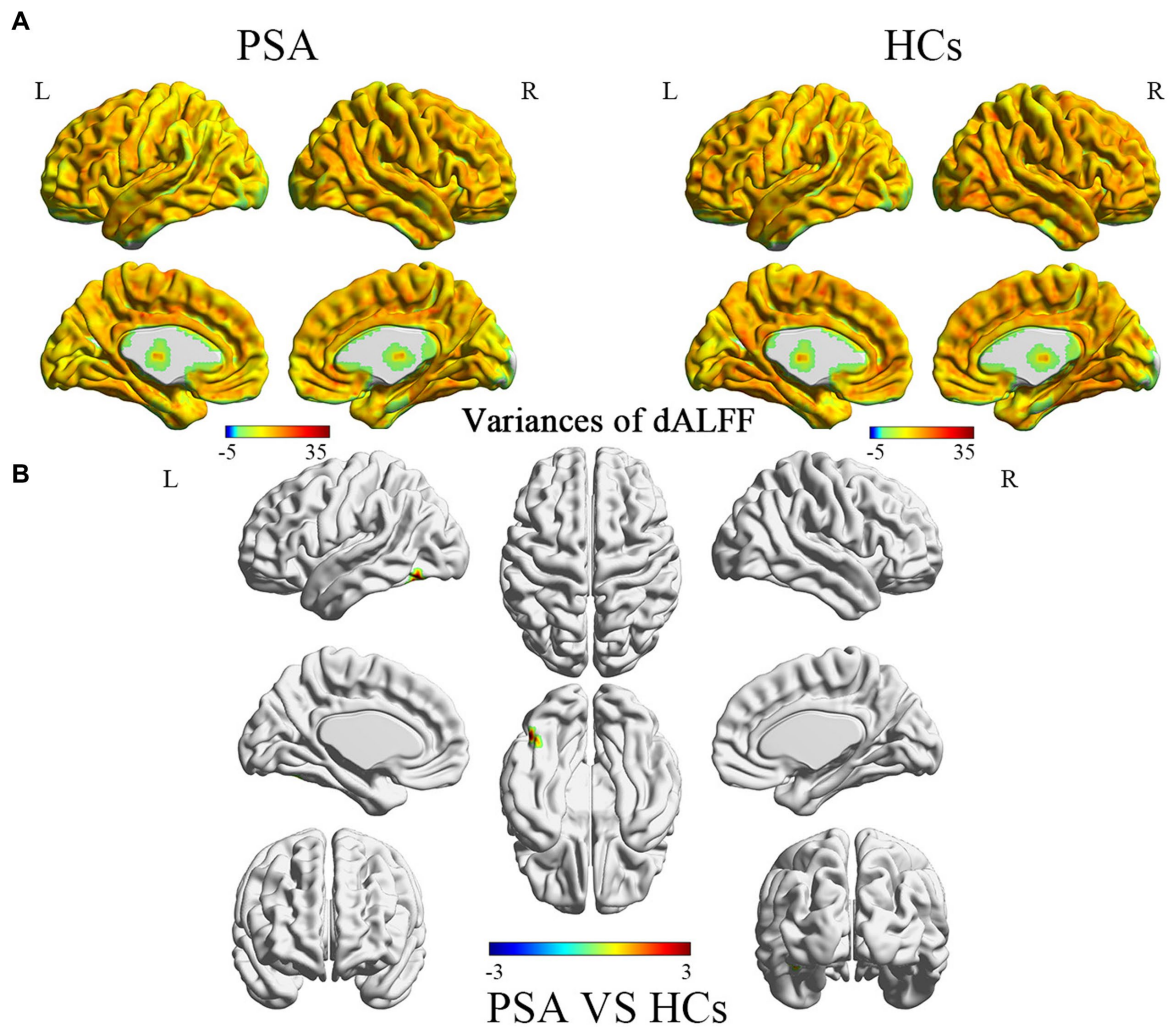
**(A)** Variances of dALFF value in different brains between PSA and HCs group. **(B)** Brain regions exhibiting significant differences in dALFF variability between the PSA and HC groups (*p* < 0.001, corrected by GRF). Warm colors represent the significance of higher variances of dALFF values, while the cool colors represent the significance of lower variances of dALFF values for the group comparisons. PSA, post-stroke aphasia; HCs, healthy controls.

Differences between dALFF variability between the PSA and HC groups are shown in Table 2 and Figure 2. In the PSA group, the brain regions exhibiting increased dALFF variability were mainly located in the left superior cerebellum (CRBLCrus1.L and CRBL6.L), in the left inferior temporal gyrus (ITG.L), and in the left fusiform gyrus (FFG.L).

**Table 2:** Regions exhibiting between-group differences in dALFF variability.

Clusters	Cluster size	Brain regions/Brain networks	Peak MNI coordinates	*t*-values
			x y z	
Cluster 1	30	CRBLCrus1.L / CBN	−39 −63 −21	5.1103
		ITG.L / FTPN		
		FFG.L / FTPN		
		CRBL6.L / CBN		

The threshold was set at *p* < 0.001 (GRF corrected); MNI, Montreal Neurological Institute space; CBN, cerebellar network; FTPN, fronto-temporo-parietal network; CRBLCrus1.L, left superior cerebellum; ITG.L, left inferior temporal gyrus; FFG.L, left fusiform gyrus; CRBL6.L, left superior cerebellum.

### Clustered dALFF states

**Figure 3:**
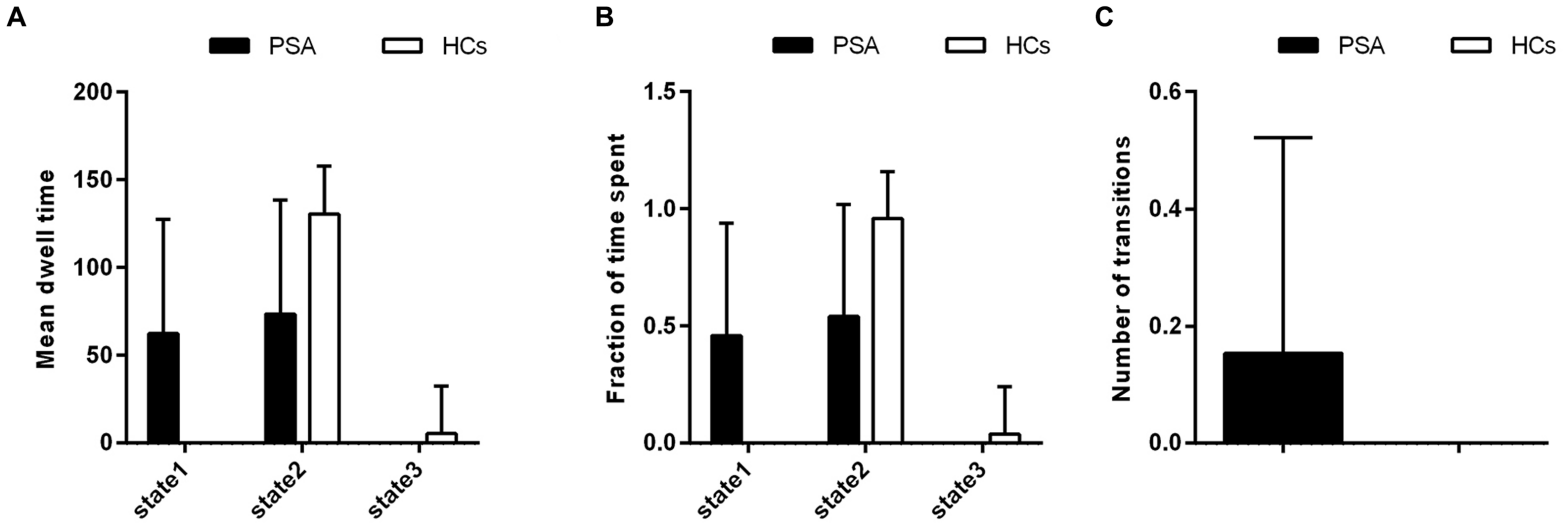
Group differences in regard to metrics of the dALFF states. Differences between the PSA and HC groups were observed in regard to: **(A)** mean dwell time (MDT), **(B)** the fraction of time for the two states, and **(C)** the number of transitions (NT). PSA, post-stroke aphasia; HCs, healthy controls.

Three dALFF states were identified among all subjects according to the k-means clustering method. Differences in the metrics of the three states between the PSA and HC groups are shown in Figure 3. In the PSA group, states 1 and 2 were identified, and the two dALFF states share a similar proportion. However, in the HC group, states 2 and 3 were identified, and state 2 accounted for a greater proportion of the two states. Furthermore, the PSA group exhibited a significantly higher F and longer MDT in state 1, yet a lower F and shorter MDT in the other two states. Moreover, the NT between the two states in the PSA group was greater than that in the HC group (Figure 3C).

### Validation results

The results obtained from using window lengths of 30TRs (1TR) and 40TRs (2TR) were similar to the main results obtained above (Supplementary Figure Supplementary Figures S1–S3 and Supplementary Figure Supplementary Tables S1, S2). In addition, in the 30 TRs window length analysis, increased dALFF variability was observed in the left postcentral gyrus (PoCG.L) (Supplementary Figure Supplementary Figure S1 and Supplementary Figure Supplementary Table S1).

## Discussion

This study explores the changes of brain functional activities during acute PSA via using the sliding-window dALFF method from two aspects: dALFF variability and dynamic patterns of ALFF. The results showed abnormal functional activity was detected in the left superior cerebellum, the left inferior temporal gyrus, and in the left fusiform gyrus of PSA patients. At the brain network level, mainly in the cerebellar network (CBN) and left fronto-temporo-parietal network (FTPN). Using k-means clustering, three dALFF states were identified among all subjects. There are differences in state distribution and state properties between PSA and HC groups. The number of transitions between the two dALFF states was higher in the PSA group than in the HC group. Taken together, these findings provide valuable insights into the brain dysfunction that occurs during the acute phase (6.00 ± 3.52 days) of PSA.

### Differences in dALFF variability

It has been widely accepted that the cerebellum is primarily a modulator of motor functions, including diadochokinesia, tonus, coordination, and motor speech production (De Smet et al., 2013). However, over the past few decades, accumulating evidence from neuroanatomic, neurophysiologic, neuroimaging, and clinical studies have updated our understanding of the role of cerebellum. A large number of studies have proposed that the cerebellum plays important roles in cognitive (Ashida et al., 2019), affective (Adamaszek et al., 2017), and language functions (Fiez, 2016; Ashida et al., 2019). In addition, some researchers have proposed that the cerebellum is related to multiple aspects of language function (De Smet et al., 2013; Mariën and Borgatti, 2018). The latter include: motor speech production, verbal fluency, lexical retrieval, grammatic/syntactic processing, reading, and writing. In the present study, increased dALFF variability was observed in the CBN in the PSA group. However, in previously conducted static studies, no alternations were observed in the CBN in PSA patients. These results suggest that dALFF can better capture changes in spontaneous neural activity in acute PSA patients. At the same time, we hypothesize that these changes in functional activity in the CBN may be related to the spontaneous functional recovery of language during acute PSA. And our future studies will focus on enrolling a larger cohort to verify the reliability of this finding.

It is well-established that the left FTPN is closely connected with language, and it covers the left middle temporal gyrus, left superior temporal gyrus, ITG.L, FFG.L, as well as other language ability-related regions. As we all know, the left middle temporal gyrus is critical in lexical and semantic aspects (Python et al., 2018), especially in naming. Moreover, the left superior temporal gyrus is the center for comprehension and planning of words (Javed et al., 2022). In the present study, increased dALFF variability was observed in the ITG.L, and this may be related to functional reorganization of the peripheral region of language-related regions. In addition, increased dALFF variability was also observed in the FFG.L. It has been proposed that the FFG.L is related to reading (Chetail et al., 2018; Gerrits et al., 2019). Combined with previous research (Geranmayeh et al., 2016; Billot et al., 2022), we considered the observed increased variability of local functional activities in the left FTPN to potentially represent a “compensatory activation” mechanism that promotes functional recovery of language in the acute phase of PSA.

In the 30 TRs window length analysis that was conducted, increased dALFF variability in the left postcentral (PoCG.L) was observed. Previous studies have reported that the PoCG.L is related to language function, and mainly manifests as a “compensatory” activation after language dysfunction (Dickens et al., 2019; Yi et al., 2019). Thus, it is reasonable to speculate that the increased dALFF variability observed in the PoCG.L is a compensatory mechanism of functional recovery of language.

### Differences in metrics of the dALFF states

There were three dALFF clustering states identified in the resting state between the PSA and HC groups. In the HC group, state 2 and state 3 were identified, and state 2 accounted for the greater proportion of the two states. These results suggest that state 2 may represent a major brain activity pattern in healthy individuals. However, in the PSA group, state 1 and state 2 were identified, and the two dALFF states share a similar proportion. These differences in brain activity patterns suggest that disturbances in functional activity occur after PSA. Typically, MDT, F, and NT are used as parameters in dynamic pattern analysis to describe state properties (Allen et al., 2014; Dong et al., 2022). These properties represent brain functional activity and may be reconfigured during illness (Marusak et al., 2017; Fu et al., 2018). In the present study, MDT and F were increased in state 1 in the PSA group, while MDT and F were reduced in the state 2, compared with the HC group. We hypothesize that this phenomenon is due to an imbalanced distribution of brain functional activity. Furthermore, the NT of dALFF were increased in the PSA group. It has been suggested that the NT between different states enables multiple brain regions to gain flexibility. However, Zhao et al. (2021) propose that an increase in NT is related to a lower efficiency of information flow in the brain network. In our study, we believe that the dysfunction of language network leads to lower efficiency of information flow, and then the NT increased in the PSA group. This result also hinted that the whole brain integration of language network is abnormal.

## Limitations and future directions

There are several limitations associated with the current study which should be considered. First, the selection of the length of the sliding window used is still in dispute. However, the results of verification analysis using window lengths of 30 TRs (step size 1TR) and 40 TRs (step size 2TRs) are basically consistent with the main results. This suggests that our results are relatively reliable. Second, all of the patients included in the present study exhibited mild aphasia. Third, the sample size of the present study was small. Therefore, future studies should enroll a greater number of patients and healthy subjects to confirm the present findings. The method of dALFF provide valuable insights into exploring changes in brain functional activity. And it would enable elucidating pressing questions, such as: are dynamic neural activity measures predictive of future outcomes, especially in regard to treatment effects, including medical treatment, repetitive transcranial magnetic stimulation (rTMS) and transcranial direct current stimulation (tDCS)? This will be the focus of future research.

## Conclusion

In summary, increased variability of local functional activities in multiple language-related regions of the brain which is observed in the present study may be related to spontaneous functional recovery of language. What’s more, abnormal distribution of state properties was caused by disturbance of cerebral functional activities during the acute phase (6.00 ± 3.52 days) of PSA. Further understanding of the recovery mechanism(s) of PSA is still needed and vital to establish guidelines for effective therapy.

## Data availability statement

The original contributions presented in the study are included in the article/Supplementary Figure Supplementary material, further inquiries can be directed to the corresponding author.

## Ethics statement

The studies involving human participants were reviewed and approved by the IRB of Yantai Yuhuangding Hospital Affiliated to Qingdao University. The patients/participants provided their written informed consent to participate in this study. Written informed consent was obtained from the individual(s) for the publication of any potentially identifiable images or data included in this article.

## Author contributions

LX wrote the manuscript. LX, TC, and ZL conceived of the idea and performed the literature review. HW and ZS performed the data analysis. ML, RL, and LJ contributed to the data collection. All authors revised the manuscript and approved the final version.

## Funding

This work was partially supported by grants from the Yantai Science and Technology Plan Project (2021YD033 and 2018SFGY092).

## Conflict of interest

The authors declare that the research was conducted in the absence of any commercial or financial relationships that could be construed as a potential conflict of interest.

## Publisher’s note

All claims expressed in this article are solely those of the authors and do not necessarily represent those of their affiliated organizations, or those of the publisher, the editors and the reviewers. Any product that may be evaluated in this article, or claim that may be made by its manufacturer, is not guaranteed or endorsed by the publisher.

## Footnotes

^1^ http://www.restfmri.net/forum/DPARSF
