# Supplementary material for: Dynamic alterations of spontaneous neural activity in post-stroke aphasia: a resting-state functional magnetic resonance imaging study
Source: Front Neurosci. 2023 May 11;17:1177930. doi: 10.3389/fnins.2023.1177930 (PMC10213748; doi:10.3389/fnins.2023.1177930)
Supplement: Supplementary file 1 [file Table_3.DOCX]

**TABLE S1 |** Regions exhibiting between-group differences in dALFF variability (Window width = 30TR & Step = 1TR)

| Clusters | Cluster size | Brain regions/Brain networks | Peak MNI coordinates | t- values |
| --- | --- | --- | --- | --- |
|  |  |  | x y z |  |
| Cluster1 | 29 | CRBLCrus1.L/CBN  ITG.L/FTPN  FFG.L/FTPN  CRBL6.L/CBN | -42 -63 -21 | 4.5353 |
| Cluster2 | 31 | PoCG.L | -21 -36 78 | 4.2701 |

The threshold was set at a *P* < 0.001(GRF corrected); MNI, Montreal Neurological Institute space; CBN, Cerebellar network; FTPN, fronto-temporo-parietal network; CRBLCrus1.L, left superior cerebellum; ITG.L, left inferior temporal gyrus; FFG.L, left fusiform gyrus; CRBL6.L, left superior cerebellum; PoCG.L, left postcentral gyrus.
